# The Role of Pro-Inflammatory and Regulatory Signaling by IL-33 in the Brain and Liver: A Focused Systematic Review of Mouse and Human Data and Risk of Bias Assessment of the Literature

**DOI:** 10.3390/ijms21113933

**Published:** 2020-05-30

**Authors:** Nika Zharichenko, Dolores B. Njoku

**Affiliations:** 1 Department of Anesthesiology and Critical Care Medicine Johns Hopkins University, Baltimore, MD 21287, USA; nzharic1@jh.edu; 2 Department of Anesthesiology and Critical Care Medicine, Johns Hopkins University, Baltimore, MD 21287, USA; 3 Department of Pediatrics, Johns Hopkins University, Baltimore, MD 21287, USA; 4 Department of Pathology, Johns Hopkins University, The Charlotte R. Bloomberg Childrens Center, 1800 Orleans Street, Suite 6349D, Baltimore, MD 21287, USA

**Keywords:** IL-33, inflammation, up-regulation, down-regulation, brain, central nervous system, liver, hepatitis

## Abstract

Interleukin (IL)-33 is a member of the IL-1 family of proteins that have multiple roles in organ-specific inflammation. Many studies suggest diagnostic and therapeutic implications of this cytokine. Many studies have reported pro-inflammatory roles for IL-33 in innate immune responses involving the heart and lung. Recent studies also describe pro-inflammatory and regulatory roles for IL-33 in the pathogenesis of brain and liver disorders in addition to regulatory roles for this cytokine in the heart and lung. In this focused systematic review, we will review the literature regarding pro-inflammatory and regulatory effects of IL-33 in the brain and liver. We will also assess the potential risk of bias in the published literature in order to uncover gaps in the knowledge that will be useful for the scientific community. We utilized guidelines set by preferred reporting items for systemic reviews and meta-analyses. The electronic database was PubMed. Eligibility criteria included organ-specific inflammation in mice and humans, organ-specific inflammation in the central nervous and hepatic systems, and IL-33. Outcomes were pro-inflammatory or regulatory effects of IL-33. Risk of bias in individual studies and across studies was addressed by adapting the Cochrane Rob 2.0 tool. We discovered that a source of bias across the studies was a lack of randomization in human studies. Additionally, because the majority of studies were performed in mice, this could be perceived as a potential risk of bias. Regarding the central nervous system, roles for IL-33 in the development and maturation of neuronal circuits were reported; however, exact mechanisms by which this occurred were not elucidated. IL-33 was produced by astrocytes and endothelial cells while IL-33 receptors were expressed by microglia and astrocytes, demonstrating that these cells are first responders for IL-33; however, in the CNS, IL-33 seems to induce Th1 cytokines such as IL-1β and TNF-α chemokines such as RANTES, MCP-1, MIP-1α, and IP-10, as well as nitric oxide. In the liver, similar risks of bias were determined because of the lack of randomized controlled trials in humans and because the majority of studies were performed in mice. Interestingly, the strain of mouse utilized in the study seemed to affect the role of IL-33 in liver inflammation. Lastly, similar to the brain, IL-33 appeared to have ST2-independent regulatory functions in the liver. Our results reveal plausible gaps in what is known regarding IL-33 in the pathogenesis of brain and liver disorders. We highlight key studies in the lung and heart as examples of advancements that likely occurred because of countless basic and translational studies in this area. More research is needed in these areas in order to assess the diagnostic or therapeutic potential of IL-33 in these disorders.

## 1. Introduction

Interleukin (IL)-33 is a member of the IL-1 family of 11 cytokines that include IL-1α and β, IL-1 RA, IL-18, IL-36α, β, and γ, IL-36RA, IL-37, and IL-38. Similar to its family members, IL-33 can both initiate and regulate inflammation [1,2]. In humans, IL-33 is expressed constitutively in barrier tissues, lymphoid organs, brain and spinal cord, liver, and embryos, and can be induced in inflamed tissue [1,2,3]. By contrast, in mice, IL-33 is expressed most abundantly in adipose tissue and in the liver [2,3].

**Figure 1:**
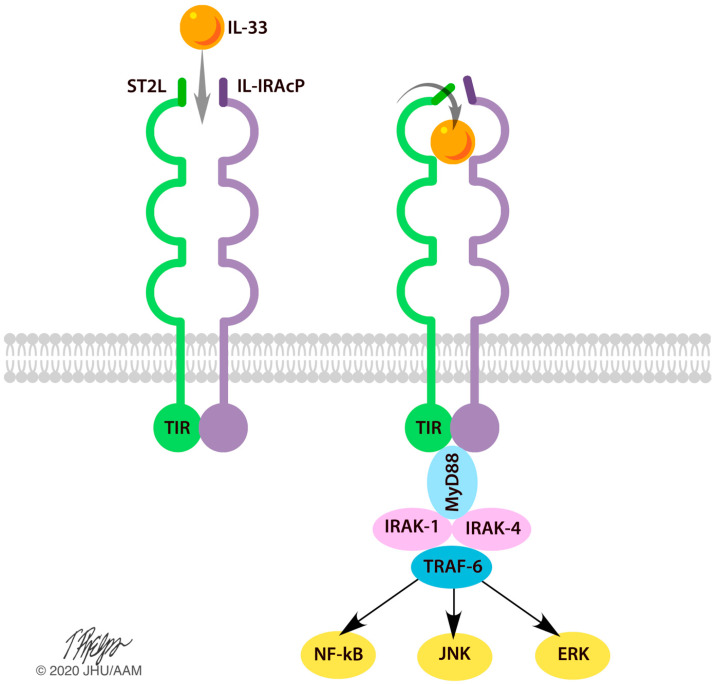
IL-33 mechanism of activation. The ST2L/IL-1RAcP receptor complex is found on the cell membrane of Th2 cells, ILC2s, and FoxP3+ Tregs. Upon binding to IL-33, this receptor complex undergoes a conformational change to allow for activation of the inflammatory response. A cascade of events unfolds, in which MyD88, IRAK-1, IRAK-4, and TRAF-6 become activated. Subsequently, NF-kB, JNK, and ERK/MAPK pathways are activated to produce a pro-inflammatory response. TIR: Toll interleukin receptor; MyD88: Myeloid differentiation primary response protein; IRAK: Interleukin receptor-associated kinase; TRAF: TNF-receptor-associated factor 6; NF-κB: Nuclear factor kappa light chain enhancer; JNK; c-Jun N-terminal kinases; ERK: Extracellular signal-regulated kinase.

**Figure 2:**
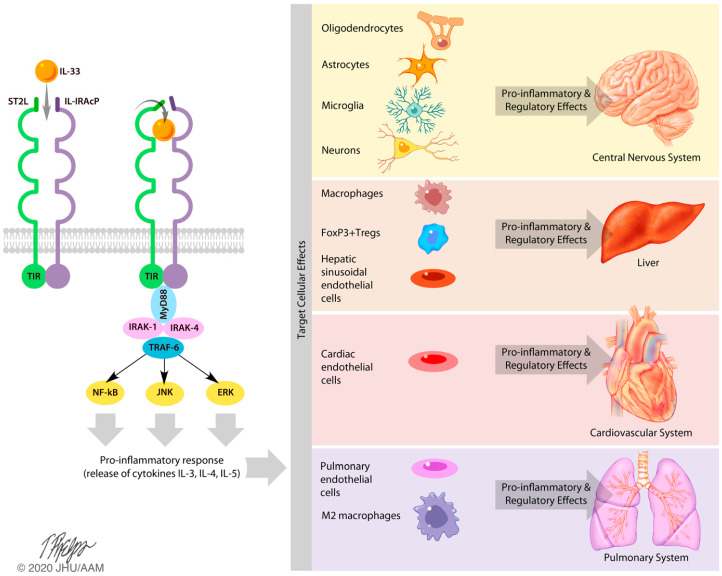
Effects of IL-33, as pro-inflammatory and regulatory, on the central nervous system, liver, cardiovascular system, and pulmonary system. Following activation of the IL-33 pathway, and subsequent activation of NF-κB, JNK, and ERK/MAPK pathways, a pro-inflammatory response is initiated. This pro-inflammatory response results in the release of cytokines, including IL-3, IL-4, and IL-5. These cytokines act on target cells within the central nervous, hepatic, cardiovascular, and pulmonary systems. Within the central nervous system, activated oligodendrocytes, astrocytes, microglia, and neurons induce pro-inflammatory or regulatory effects. In the liver, activated macrophages and hepatic sinusoidal endothelial cells (HSECs) induce pro-inflammatory effects, while FoxP3+ Tregs induce regulatory effects. In the heart, cardiac endothelial cells induce pro-inflammatory or regulatory effects. In the pulmonary system, pulmonary endothelial cells and M2 macrophages induce pro-inflammatory and regulatory effects.

Since 1980, IL-33 has been mentioned in the literature over 800 times. The majority of papers discuss the role of IL-33 in the pathogenesis of autoimmune or allergic responses. In these responses, IL-33 is described as a danger signal or alarmin that is expressed following cellular injury [1,4,5,6,7]. Once expressed, IL-33 is believed to bind to the suppression of the tumorigenicity 2 (ST2) receptor via its IL-1-like cytokine domain. IL-33/ST2 binding recruits the IL-1 receptor accessory proteins (IL-1RAcP), forming a heterotrimeric signaling complex that is capable of activating DNA transcription and nuclear cytokine production via nuclear factor kappa-light-chain-enhancer of activated B cells (NF-κB), serine/threonine protein kinases such as ERK/MAPK, as well as major subsequent MAPK pathways such as c-Jun N-terminal kinase (JNK) (Figure 1) [1,4]. Ultimately, these pathways promote the release of pro-inflammatory cytokines 1L-3, IL-4, and IL-5 (Figure 1) [1,8]. These cytokines are then capable of inducing inflammatory and regulatory responses in the central nervous system, the liver, the heart, and the lungs (Figure 2).

IL-33 can be either pro-inflammatory or regulatory depending on the organ involved or disease process, as well as the affected cell type and the stimulus that evoked the IL-33 immune response [8,9]. Much attention has been given in the literature to the pro-inflammatory role of IL-33 via ST2 receptor engagement in the innate immune system. However, the ST2 receptor is expressed on many types of cells including Th2 cells, group 2 innate lymphoid cells (ILC2s), regulatory T cells, endothelial cells, dendritic cells, macrophages, and M2 macrophages (Figure 2) [4]. Endothelial cells line the inner surface of blood vessels and organs, making them likely IL-33 first-responders in immune responses. These endothelial cells on barrier surfaces participate in host defense against injury or extracellular pathogens [8]. Dendritic cells in their role as antigen-presenting cells make them likely IL-33-promoted bridges between the innate and adaptive immune system. Macrophages, in their scavenger roles of engulfing foreign substances and pathogens, could also function as IL-33 first responders [8]. In this way, prior studies demonstrate that IL-33 can promote macrophage polarization.

Significant roles in maintaining tissue homeostasis have also been attributed to IL-33. Prior studies show that IL-33 has an important role in the induction and maintenance of FoxP3+ T-cells (Tregs) and ILC2s [10]. Subsets of Tregs, including those found in adipose tissue, require IL-33 for maintenance and function [11]. IL-33 also activates ILC2s, which are involved in the proliferation and differentiation of CD4+ T cells, as well as the proliferation of type 2 cytokines IL-5 and IL-13 [12]. Thus, in addition to pro-inflammatory roles, IL-33 has regulatory roles in promoting tissue repair and maintaining tissue homeostasis [11].

In spite of these studies delineating pro-inflammatory and regulatory roles for IL-33, very few studies focus on the role of IL-33 in the central nervous system and the liver. To this point, the last comprehensive review that included what we knew at the time about IL-33 and included the brain and the liver was in 2016 [4]. Moreover, in many instances when human studies were performed, the study populations needed more definition in order to comprehend the role of IL-33. Additionally, many rodent studies utilized C57BL/6, BALB/c, or unspecified mouse models without comparing responses between strains. The aim of this focused systematic review is to analyze the literature that examines the effects of the role of IL-33 in organ-specific inflammation within the central nervous system and the liver. We will evaluate research published from 1987 to 2020. We will exclude case reports at the outset due to their limited external validity. Our primary aim is to map the literature on IL-33 in the pathogenesis of brain and liver disorders in order to identify key concepts and uncover plausible gaps in the knowledge. While the cardiovascular and pulmonary systems are not the original focus of our research, we have included these systems because we felt that they demonstrate sizable differences in the amount of data reported when compared to the central nervous and hepatic systems. In addition, these comprehensive basic and translational studies investigating the cardiovascular and pulmonary systems likely contributed to a greater understanding of the role of IL-33 in these organs. Our secondary aim is to promote more research in these areas that will assess the diagnostic or therapeutic potential of IL-33 in brain and liver disorders that will ultimately be utilized to inform patient practice.

## 2. Methods

### 2.1. PRISMA Criteria

We followed the guidelines set by PRISMA (Preferred reporting items for systematic reviews and meta-analyses) in constructing this review [13]. The relevant checklist items from PRISMA were included. Items related to meta-analysis were excluded. As recommended by Prospero, the international prospective register of systematic reviews, we plan to register this protocol once peer review has been completed.

### 2.2. Protocol and Registration

This protocol includes a focused systematic review of health research studies including human subjects, as well as mouse subjects. We will register this protocol once peer review has been completed.

### 2.3. Eligibility Criteria

Our eligibility criteria included health research, as well as mouse research, involving IL-33 and organ-specific inflammation in the brain and liver. This search was from 1987 until the present. Human studies that were considered included randomized control trials, clinical studies, clinical trials, comparative studies, observational studies, reviews, systematic reviews, and controlled clinical trials. Mouse research had to have direct relevance to human health. The observed outcomes were pro-inflammatory effects or regulatory effects of IL-33 in the brain and liver.

### 2.4. Information Sources

Studies were identified by searching PubMed and browsing reference lists of selected articles. Limitations were placed on language, including only articles published in English. The last search was run on April 16, 2020.

### 2.5. Search

Relevant keywords were searched in various combinations. The keywords included, but were not limited to: IL-33, interleukin-33, brain, central nervous system, liver, hepatic system, cardiovascular system, heart, cardiac, pulmonary system, lungs, regulatory, pro-inflammatory, FoxP3, and Tregs. Table 1 provides a more detailed list of the keywords used in the search strategy and inclusion criteria in terms of population of interest, intervention, comparison group, and outcome. Searches involved the following terms: (IL-33 OR interleukin-33), OR (IL-33 AND central nervous system), OR (IL-33 AND brain), OR (IL-33 AND liver), OR (IL-33 AND hepatic system), OR (IL-33 AND cardiovascular system), OR (IL-33 AND heart), OR (IL-33 AND cardiac), OR (IL-33 AND pulmonary system), OR (IL-33 AND lungs). The search was not limited by date of publication but was limited by inclusion of both IL-33 and the organ-system in question. The selection process was based on title, abstract, and full text. In addition, the search was limited by studies that directly mentioned the effects of IL-33, specified organs, and including our outcome of interest: Pro-inflammatory and regulatory. Additionally, the reference lists for each article also searched for supplementary papers. These additional articles were selected in the same way as the primary publications.

### 2.6. Study Selection

**Figure 3:**
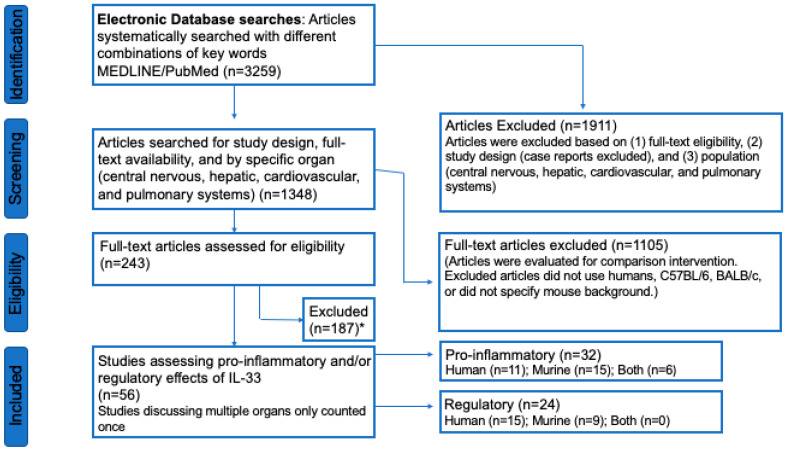
PRISMA Study Selection Flow Diagram. The PRISMA Flow Diagram demonstrates each phase of the search strategy in which articles were evaluated. During each phase, articles were excluded based on our defined criteria. The last phase of exclusion resulted in a final collection of articles, which were then divided into a pro-inflammatory or regulatory. These articles were then further broken down by population, including humans, mice, and both. * The majority of full-text articles were excluded because they were review articles published prior to December 31 2017, IL-33 was not a main focus of the article, or the article was not available in English.

The study selection is indicated in Figure 3 by the PRISMA flow diagram [13]. An electronic database search of PubMed/MEDLINE with several key terms in various permutations was performed. During the first stage, the articles were scanned for appropriate titles/abstracts containing IL-33 and an association to the specific organs (CNS, liver, cardiovascular system, pulmonary system). The articles that were deemed appropriate were evaluated further, while articles that did not meet inclusion criteria were excluded. During this stage, articles were excluded based on the population and comparison intervention, including articles that mentioned a population other than humans or C57BL/6 and BALB/c mice, or did not focus on the specified organs (CNS, liver, cardiovascular system, pulmonary system).

The next stage removed additional articles not meeting inclusion criteria. This stage continued to evaluate the population and comparison intervention based on full-text searches. The population of interest consists of humans and/or C57BL/6 and BALB/c mice. All other articles discussing different animal models were excluded. The desired comparison intervention was the specific organ systems of interest (CNS, hepatic, cardiovascular, pulmonary), as well as the comparison between murine models and human models, if applicable. Articles were included if the full-text article elaborated on the information included in the abstract to clarify the population and comparison. In addition, case reports were excluded due to the reduced level of generalizability.

In the final stages, we identified which full-text articles included information on the pro-inflammatory or regulatory nature of the effect of IL-33 at an organ-specific level. At this stage, the search of population and comparison intervention continued to be refined, while the exclusion criteria focused on the desired outcome. Articles that did not directly discuss the effects of IL-33 in the scope of each organ system were excluded. Articles that discussed multiple organs were only counted once as either pro-inflammatory or regulatory as determined by relevance. Articles addressing the effects of the IL-1 family of cytokines in the organ systems were included to understand and analyze various mechanisms of action. Other immune factors were also included in this review, particularly the NLRP3 inflammasome, soluble ST2 receptor, ST2 receptor, FoxP3+ Tregs, IL-10, and ERK/MAPK pathway. Articles without full text availability were excluded from this review.

### 2.7. Data Collection Process

Information was extracted from each of the selected studies to provide summative evidence on the pro-inflammatory or regulatory effects of IL-33 within each organ system, as well as between humans and mouse models (C57BL/6 and BALB/c).

### 2.8. Data Items

Information was extracted for each included study on: (1) The pro-inflammatory effects of IL-33; (2) the regulatory effects of IL-33; (3) the organ-specific effects, including the central nervous system, liver, cardiovascular system, and pulmonary system, and effects such as pro-inflammatory, regulatory, or a combination of the two within an organ system.

### 2.9. Risk of Bias in Individual Studies

The Cochrane RoB 2.0 tool is commonly used for meta-analysis of randomized clinical trials. We adapted this tool to perform a literature analysis. The risk of bias was assessed in individual studies using the Cochrane RoB 2.0 tool [13]. We analyzed the collected data for selection bias, confirmation bias, publishing bias, observation bias, and reporting bias. We assessed the selection of the reported results, measurement of the stated outcomes, missing outcomes data, deviation from the intended result, and the randomization process. Selection bias in the studies analyzed was defined as the identification of a group of data for analysis in a way that interfered with randomization and proper representation of the study population. The Cochrane RoB 2.0 tool was used to evaluate selection bias by assessing if the allocation of data was random and if any baseline difference between groups could suggest a problem with randomization. Confirmation bias in the analyzed studies was defined as a tendency to interpret information or results in a way that confirmed prior preconceptions. An example of confirmation bias would be a manuscript that only acknowledges proinflammatory roles for IL-33, while the results could also represent regulatory roles for this cytokine. The Cochrane RoB 2.0 tool was used to evaluate confirmation bias by searching for deviations from intended goals stated in the aims of each article that could be demonstrated by interpretation of the data in a way that confirmed prior preconceptions. Publication bias was defined as when the outcome of a study influenced the decision whether to publish it. An example could be the wealth of data on proinflammatory but not regulatory roles for IL-33. The Cochrane RoB 2.0 tool was used to evaluate publication bias by evaluating the potential presence of missing outcome data that could represent an alternative conclusion. Observation bias was defined as the tendency to see what we expect to see. An example of observation bias would be a manuscript that only focused on the proinflammatory roles of IL-33 and did not include the regulatory roles although the evidence might suggest that they were present. The Cochrane RoB 2.0 tool was used to evaluate observation bias by assessing the measurements used to determine the outcome and whether the measurements were appropriate, and could have differed between groups. Reporting bias was defined as selective reporting of some outcomes that appeared to depend on the nature and direction of the results. Thus, proinflammatory results for IL-33 were reported more than regulatory results. The Cochrane RoB 2.0 tool was used to evaluate reporting bias by evaluating if the selection of the reported result was in accordance with the data presented.

### 2.10. Summary Measures

The primary outcomes of interest were the pro-inflammatory or regulatory effects of IL-33 in organ-specific inflammation. Risk ratios and differences between means were not analyzed.

### 2.11. Synthesis of Results

Results were synthesized based on the measures reported in descriptive and mechanistic studies. Descriptive studies included the intervention and a detailed account of the outcome. Mechanistic studies involved using the intervention to explore mechanisms of action, as well as adding a treatment to examine effects of the treatment on the intervention. Mechanistic studies were our goal in which IL-33 was investigated in vitro or in vivo; however, our preference was in vivo.

### 2.12. Risk of Bias across Studies

The risk of bias was assessed across studies using the Cochrane RoB 2.0 tool [13]. Similar parameters were used to assess risk of bias across studies as in individual studies.

### 2.13. Additional Analyses

No additional analyses were performed.

## 3. Results

### 3.1. Study Selection

Utilizing our inclusion and exclusion criteria, 3259 studies were deemed eligible for assessment (Figure 3). Of these articles, 1911 were excluded because they did not focus on the CNS, hepatic, cardiovascular, or pulmonary systems, or because populations other than humans or C57BL/6 and BALB/c mice were studied. In this section, case reports were excluded. Further, 1348 articles were deemed appropriate for further analysis. Of these, 1105 articles were removed for similar reasons: Because they did not include C57BL/6 or BALB/c mice, did not specify the strain of mouse utilized, or were not performed in humans. Of the remaining 243 articles, we identified 56 full-text articles that included information on the pro-inflammatory or regulatory effects of IL-33 at an organ-specific level and excluded 187 articles that did not meet this criterion. Of these 56 articles, 32 included data regarding the pro-inflammatory effects (CNS, *n* = 10; hepatic, *n* = 8; cardiovascular, *n* = 5, pulmonary, *n* = 9) and 24 included data regarding the regulatory effects (CNS, *n* = 3; hepatic, *n* = 1, cardiovascular, *n* = 10, pulmonary, *n* = 10). Of the articles discussing the pro-inflammatory effects, 11 utilized human subjects, 15 utilized a murine model, and 6 utilized both human and murine models. Within the articles discussing the regulatory effects of IL-33 in organ-specific inflammation, 15 used human subjects, and 9 used a murine model.

### 3.2. Effects of IL-33 on the Central Nervous System

**Figure 4:**
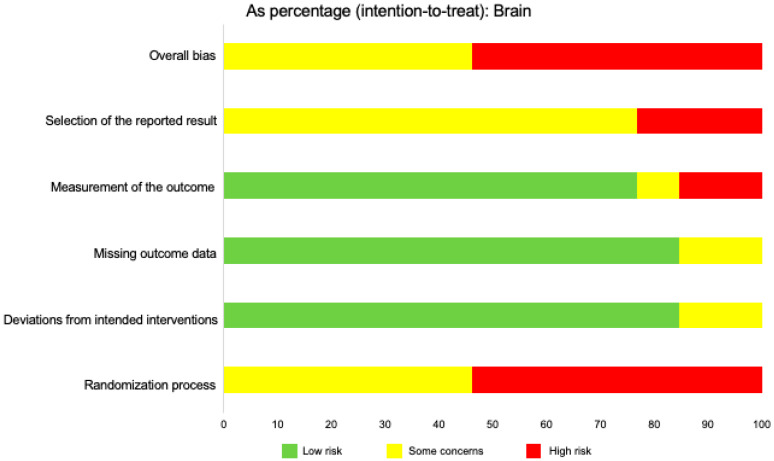
Risk of bias across studies focusing on the brain, in both mice and humans. This figure displays the relative percentages of intention-to-treat data of assessed risk of bias in the brain in human and mouse studies. The overall bias was determined using five categories: Selection of the reported result, measurement of the reported outcome, missing outcome data, deviations from intended interventions, and randomization process. Risk was determined as “low risk,” “some concerns,” or “high risk,” based on the Cochrane RoB 2.0 tool algorithm. The data for this figure included 14 articles.

We used the risk of bias tool even though we were analyzing both human and mouse studies. There was a noticeable paucity of randomized control trials. We selected intention to treat in order to detect inadvertent reporting or analysis bias in our studies. In order to determine the overall risk of bias, we assessed the selection of the reported results, measurement of the stated outcomes, missing outcomes data, deviation from the intended result, and the randomization process. In assessing the risk of bias in studies regarding IL-33 and the central nervous system, we discovered that the overall risk of bias for all human and mouse studies exposed some concerns (46.2%) or high risk of bias (53.8%) (Figure 4). The greatest contributor to the risk of bias was the lack of randomization. Additionally, selection of the reported results demonstrated some concerns (76.9%) and high risk of bias (23.1%). Measurement of the stated outcomes demonstrated low risk of bias (76.9%) with the remaining 23.1% demonstrating some concerns or high risk of bias. When analyzing missing outcomes data and deviation from the intended result, we found a primarily low risk of bias in our reported studies (84.6%) with the remaining 15.4% demonstrating some concerns. When assessing articles for pro-inflammatory or regulatory roles for IL-33, in mice, we discovered that the majority of the articles investigated pro-inflammatory roles (*n* = 15), while the minority of articles investigated regulatory roles for this cytokine (*n* = 9). We found that similar numbers of articles met criteria in human studies when considering pro-inflammatory and regulatory roles (*n* = 11, *n* = 15, respectively).

IL-33 and ST2L/1RAcP are highly expressed in brain tissues [14]. IL-33 via the ST2 receptor is thought to have a role in neuro-inflammatory diseases [15]. In the central nervous system (CNS), IL-33 is constitutively expressed in glial cells such as oligodendrocytes and astrocytes (Table 2) [1,16]. Innate signals via pathogen-associated molecular patterns (PAMPs) increase IL-33 mRNA and protein expression within these glial cells [1,15]. IL-33 also increases expression of Toll-like receptor (TLR) ligands.

Microglia are important IL-33 first responders in the CNS (Table 2) [16]. Although IL-33 is not produced by microglia or neurons, IL-33 receptors have been demonstrated on these cells [16]. Furthermore, IL-33 engagement by microglia enhances the production of chemokines such as RANTES, MCP-1, MIP-1α, and IP-10, as well as nitric oxide [16]. Interestingly, IL-33 engagement by microglia induces cytokines such as IL-1β and TNF-α, as well as regulatory cytokines, such as IL-10, and expected Th2 cytokines such as IL-13 [16]. This finding suggests to us that in the CNS, IL-33 can induce Th1 cytokines in addition to the classical Th2 response that has been associated with IL-33.

The abundance of IL-33 in the CNS could suggest that IL-33 has a role in the regulation and maturation of neuronal circuits. IL-33 is expressed in various brain regions including the corpus callosum and secondary motor cortex, the medial prefrontal cortex, the periventricular hypothalamic nucleus, and the amygdala (Table 3). As IL-33 is expressed in areas of the limbic system such as the amygdala, it is not surprising that IL-33 deficiency has been associated with anxiety-related behaviors [1]. In one study, IL-33-deficient C57BL/6 mice demonstrated anxiety-related behaviors measured by the open field test that is commonly used to assess locomotor activity levels, as well as anxiety and willingness to explore. However, exact mechanisms of how IL-33 alters neuronal circuits were not identified. In addition, results were not measured in IL-33-deficient BALB/c mice. This latter statement is important because BALB/c mice demonstrate increased levels of anxiety-related behaviors assessed by the open field and elevated plus maze behavioral tests when compared to C57BL/6 mice [17]. BALB/c mice also demonstrate elevated stress-induced plasma cortisol levels following acute stressors when compared to C57BL/6 mice, which can skew responses to IL-33 generated in the limbic system [17]. However, there are no differences in glucocorticoid receptor mRNA expression, specifically in the hippocampus and paraventricular nucleus, following acute stressors between BALB/c and C57BL/6 mice [17]. Thus, it is not known whether IL-33 affects the hypothalamic-pituitary axis. Moreover, whether IL-33 has a direct or downstream effect on anxiety-related behavior is not known.

In human studies, IL-33 and ST2L/1RAcP have been suggested as novel biomarkers or potential therapeutic targets in diseases affecting the CNS [14]. Higher concentrations of IL-33 in cerebrospinal fluid have been associated with depression [18]. Moreover, IL-33 has been associated with Alzheimer’s disease, multiple sclerosis, schizophrenia, CNS injury, CNS parasitic infection, and glioma [14,19,20,21]. Previous studies have shown that soluble ST2 (sST2) levels are significantly associated with pro-inflammatory responses, as well as severity and prognosis of disease, in traumatic brain injury [22]. Elevated serum sST2 levels have been detected in acute ischemic stroke [23]. However, without randomized controlled studies of IL-33 or sST2, it is difficult to determine the diagnostic potential of these markers.

### 3.3. Effects of IL-33 on the Liver

**Figure 5:**
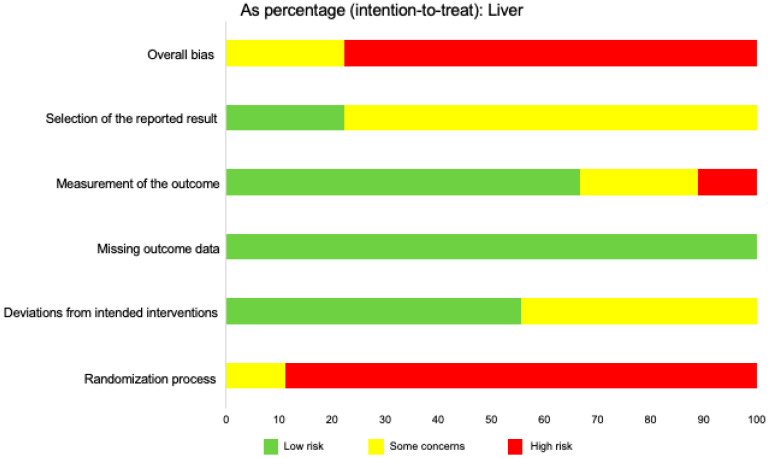
Risk of bias across studies focusing on the liver, in both mice and humans. This figure displays the relative percentages of intention-to-treat data of assessed risk of bias in the liver in human and mouse studies. The overall bias was determined using five categories: Selection of the reported result, measurement of the reported outcome, missing outcome data, deviations from intended interventions, and randomization process. Risk was determined as “low risk,” “some concerns,” or “high risk,” based on the Cochrane RoB 2.0 tool algorithm. The data for this figure included nine articles.

In assessing overall risk of bias within mouse and human studies that focused on the liver, 22.2% of studies demonstrated some concerns, while 77.8% of studies demonstrated a high risk of bias (Figure 5). Similar to studies involving the central nervous system, the greatest contributor of bias in these studies was the lack of randomization in the study designs. The only other domain that demonstrated a high risk of bias was the measurement of the reported outcome (11.1%). However, the majority of studies demonstrated a low risk of bias in this domain (66.7%) and 22.2% demonstrated some concerns. All the articles utilized in this review demonstrated a low risk of bias when analyzing missing outcomes data. When analyzing deviations from intended outcomes, 55.6% of articles demonstrated a low risk of bias while 44.4% demonstrated some concerns. Bias may have been introduced in the published literature because most studies were done in mice, and no study focused exclusively on humans. Reporting bias may have also been present in the literature similar to studies regarding IL-33 in the CNS, because the majority of studies focused on pro-inflammatory roles for IL-33 (*n* = 8) and only one focused on regulatory roles for this cytokine in the liver (*n* = 1).

The road to understanding the role of IL-33 in the liver has been complex. Pro-inflammatory responses triggered by IL-33 have been suggested in the pathogenesis of acute and chronic hepatitis and their sequelae [24]. However, regulatory roles for IL-33 have also been demonstrated in the liver when investigating effects of IL-33 on invariant natural kill T (NKT) cells and FoxP3+ Tregs. Thus, in primary biliary cirrhosis, IL-33 repairs biliary cells, but signaling is carcinogenic [25]. Furthermore, IL-33 reduces liver damage in mice with concanavalin A immune-mediated hepatitis. Cellular diversity likely affects outcomes after IL-33 expression, and this diversity is never more present than in the liver. Along these lines, a recent study demonstrated that IL-33 promotes the initiation of drug-induced hepatitis and was required for the resolution of disease through its role in the induction and function of Foxp3+ Tregs demonstrating acute and chronic pro-inflammatory and regulatory roles for IL-33, at least in mice [26]. In ischemia-reperfusion injury, dual roles for IL-33 have also been demonstrated. IL-33, via ST2, is up-regulated during ischemia-reperfusion injury in mouse models [27]. However, IL-33 down-regulates and limits liver damage and reduces inflammation via activation of NF-kB and MAPK pathways [28]. Evidence suggests that the regulatory role for IL-33 in ischemia-reperfusion injury occurs via invariant NKT cells that reduce sterile inflammation [29].

When further reviewing chronic liver diseases, we found that IL-33 has been strongly associated with liver fibrosis in mouse models [2]. IL-33 release takes on the role of hepatic fibrotic factor, which augments disease [30]. Furthermore, IL-33 along with other substances are released by the NLRP3 inflammasome, which highlights its importance in pro-inflammatory responses demonstrated in chronic inflammatory liver conditions [31]. In the liver, IL-33 is most abundant in hepatic sinusoidal endothelial cells (HSECS). In fibrosis, these activated cells promote accumulation of extracellular matrix proteins in the liver sinusoids [2]. This interaction between IL-33 and HSECs is important because, in the absence of the ST2 receptor, liver injury, inflammation, and fibrosis are reduced in addition to reduced activation of HSECs [32]. Thus, critical roles for IL-33 in activation of Th2 immune responses via cytokines IL-4, IL-6 and IL-13 have been demonstrated in the pathogenesis of HSEC proliferation and liver fibrosis in mouse models [32]. Interestingly, liver damage from fibrosis and IL-33 responses vary greatly between C57BL/6 and BALB/c mice. A recent study demonstrated using a high-fat diet that differences between these murine backgrounds are likely derived from their immune or metabolic phenotypes [33]. In this study, C57BL/6 mice developed fibrosis from a high-fat diet while BALB/c mice did not, demonstrating the importance of strain in drawing conclusions regarding the role of IL-33 in fibrosis and potentially other liver diseases [33].

No recent studies have solely focused on the role of IL-33 in liver diseases affecting patients. Landmark studies have detected IL-33 in patients with acute liver failure and in patients with hepatitis C and hepatitis B [34,35,36]. However, whether IL-33 had pathogenic or protective roles in these diseases was not completely clear. Interestingly, CNS effects such as confusion, lethargy, and loss of fine motor skills are well known in hepatic encephalopathy [37]. However, whether or not these effects are induced by IL-33 is not known.

### 3.4. Effects of IL-33 on the Cardiovascular and Pulmonary Systems

Even though effects of IL-33 on the cardiovascular and pulmonary systems are not the subject of this focused review, the focus of the current literature on these organs when compared to the numbers of investigations addressing the CNS and the liver cannot be ignored. Thus, in contrast to the CNS and the liver, the majority of recent studies investigating the effect of IL-33 on the cardiovascular system have been focused on humans rather than on mice (*n* = 9, *n* = 3, respectively). Additionally, three studies addressed the role of IL-33 in the cardiovascular system in both humans and mice. The focus on human studies is likely because of the large amount of mouse studies that have already addressed the role of IL-33 in the cardiovascular system. Even so, when we assessed the risk of bias, all studies were found to have probable or high risk of bias. Another potential risk of bias was the focus of the majority of studies on the regulatory roles of IL-33 (*n* = 10) when compared to the pro-inflammatory roles for this cytokine (*n* = 2).

In recent years, few studies focused on the role of IL- 33 in cardiovascular disease using mouse models. Using our search criteria, only three studies focused exclusively on mice, and these studies focused on regulatory roles for IL-33 in cardiovascular disease. A recent paper suggested that mast cells may have a role in up-regulating expression of IL-33 in experimental cardiac transplantation, which might suggest that targeted therapies could be developed to modulate these cells. However, there are significant differences between levels of mast cells in mice and humans, which may reduce the translatability of these findings. Even so, IL-33 is thought to have a protective role in reducing heart allograft and xenograft rejection [38]. This protective role was demonstrated with reduced immune response-related damages and suggested that IL-33 could be developed as a therapeutic approach to transplant rejection [38].

In patients, IL-33 is thought to have a key role in end-stage heart failure. Nuclear IL-33 is constitutively expressed in human adult cardiac fibroblasts, as well as human adult cardiac myocytes [39,40]. A primary mechanism of cardiac IL-33 release is due to the stretching of myofibroblasts, in order to promote cell survival [41]. In patients with non-ST-elevation myocardial infarction, sST2 has been associated with poor outcomes [42,43]. Thus, sST2 has been suggested as a potential biomarker of cellular stress and injury and as a biomarker that guides treatment for heart failure and hypertension. However, wide variations in patient levels remain problematic and hinder translation to broad clinical practice [39,43]. Even so, prior studies suggest an association between low IL-33 levels and increased disease severity while other studies demonstrate that relatively low sST2 levels were associated with increased survival in critically ill patients [44,45]. These studies suggest that high IL-33 and low ST2 may be protective; however, more studies are needed to confirm these suggestions.Similar to the heart, a large amount of data in recent years has been done with humans to investigate the effects of IL-33 on the pulmonary system. In addition, similar to the heart, the focus on human studies is likely because of the large amount of mouse studies that have already addressed the role of IL-33 in the pulmonary system. Most of our conclusions were drawn for human experimental and comparative studies, demonstrating a lack of randomized controlled trials in recent years. In assessing the risk of bias in these studies, all of the studies included in this review demonstrated probable or high risk of bias.

The IL-33/ST2 pathway plays an important role in the development and maintenance of allergic inflammatory responses and asthma [8,46,47,48]. IL-33 induces inflammation in barrier pulmonary epithelial cells and mediates Th2 responses as well as mast cell activation [44,47,49]. Airway remodeling ensues in addition to goblet cell metaplasia, enhanced mucus secretion, and smooth muscle hypertrophy. IL-33-mediated inflammatory responses may also have a role in chronic allergic diseases by contributing to steroid resistance [50].

A recent study in BALB/c mice investigated the use of dietary galacto-oligosaccharides in eosinophilic airway inflammation and airway hyper-responsiveness [51]. When comparing interventions, researchers concluded that using galacto-oligosaccharides had similar effects as budesonide, because airway hyper-responsiveness was prevented in the mice, suggesting its role in the treatment of asthmatic diseases [51]. Interestingly, it has been suggested in mouse studies that IL-33 promotes inflammation without Th2 responses, suggesting that IL-33 is functionally active in an ST2-independent manner [52]. Another study demonstrated that infection in mice, with the fungal pathogen *P. murina*, induced pulmonary system-specific production of IL-33 and M2 alveolar macrophages that were capable of mounting immune responses against *P. murina* [53].

In humans, increased levels of IL-33 have been observed in epithelial cells and sera of patients with exercise-induced asthma [49]. Although a review of the effects of IL-33 on the pulmonary system is beyond the scope of this focused review, we highlight key studies in the lung and in the heart as examples of advancements that likely occurred because of countless basic and translational studies in this area.

## 4. Discussion

### 4.1. Summary of IL-33 Effects in Mice and Humans

Pro-inflammatory and regulatory roles for Il-33 have been described in the central nervous and hepatic systems. However, there are stark differences in the depth of understanding of these roles between mice and humans. In the central nervous system in mice, microglia demonstrate pro-inflammatory roles while astrocytes, oligodendrocytes and possibly neurons demonstrate regulatory roles for IL-33 (Table 4). In the hepatic system in mice, HSECs, macrophages, and hepatic stellate cells demonstrate pro-inflammatory roles while FoxP3+ Tregs demonstrate regulatory roles for this cytokine (Table 4). In sharp contrast, in humans, designations of pro-inflammatory and regulatory roles for IL-33 are less clear where oligodendrocytes and astrocytes constitutively express IL-33, and IL-33 nuclear factor and inflammatory mediators are expressed in the cytoplasm and nucleus in liver cells (Table 5). Thus, the majority of studies included in this review have focused on data from mouse studies.

**Figure 6:**
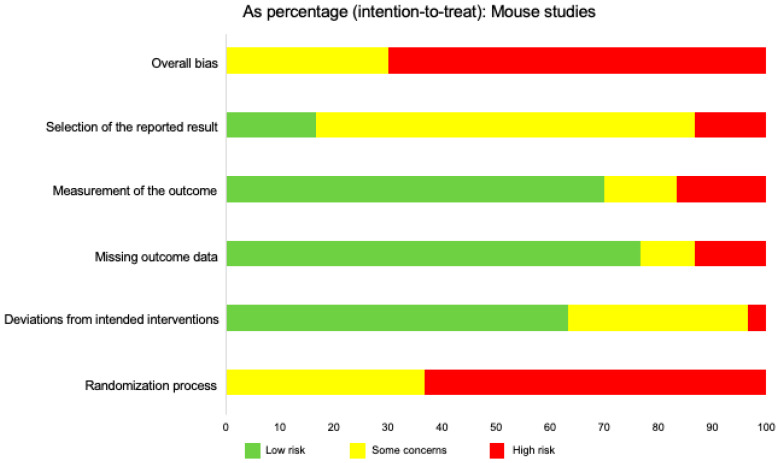
Risk of bias across all mouse studies, including brain, liver, cardiovascular, and pulmonary. This figure displays the relative percentages of intention-to-treat data of assessed risk of bias in all mouse studies. The overall bias was determined using five categories: Selection of the reported result, measurement of the reported outcome, missing outcome data, deviations from intended interventions, and randomization process. Risk was determined as “low risk,” “some concerns,” or “high risk,” based on the Cochrane RoB 2.0 tool algorithm. The data for this figure included 24 articles.

When we analyzed overall risk of bias in all mouse studies, we found that around 30% of studies demonstrated some concerns while 70% of all papers demonstrated an overall high risk of bias. Similar to the risk of bias assessments for individual organs, the lack of randomization had the single largest effect on overall bias. In contrast to studies focusing on the liver, no domain escaped high risk of bias although deviations from the intended result demonstrated the least amount of high risk of bias (<5%), and measurement of the intended outcome demonstrated the highest percentage of high risk of bias (15%) (Figure 6). Thus, most studies demonstrated a low risk of bias when assessing missing outcome data, measurement of the intended outcome, and deviations from the intended result (Figure 6).

Although it may not be possible to fully determine how bias is introduced in these mouse studies, general observations were seen. Many investigations did not specify the mouse background used in the study, which is a significant source of selection bias. In mouse models, the effects of IL-33 as pro-inflammatory or regulatory differs between organ systems. Although there is research to suggest differences in these effects in C57BL/6 when compared to BALB/c mice, research assessing this topic when trying to discern roles for IL-33 has not occurred.

Within the central nervous system, the cell types that are shown to have a pro-inflammatory role are the microglia (Table 4) [1]. Microglia are primarily involved in cell processes that cause the release of pro-inflammatory cytokines, chemokines, and oxidative stress molecules. The regulatory cells within the central nervous system include astrocytes, oligodendrocytes, and neurons. Astrocytes and oligodendrocytes function mainly as supportive cells, supporting synaptic and axonal functioning. Neurons are primarily involved in the communication of information throughout the central nervous system. However, how these cellular functions globally address IL-33 in the CNS is not clear. Similarly, in the liver, hepatic sinusoidal endothelial cells, macrophages, and hepatic stellate cells (HSCs) play a role in the pro-inflammatory effect of IL-33. By contrast, to our knowledge, only FoxP3+ Tregs have a regulatory role in the liver (Table 4).

**Figure 7:**
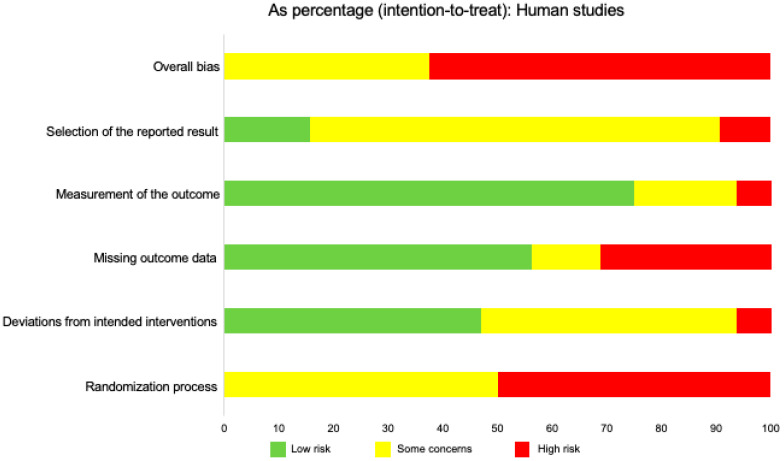
Risk of bias across all human studies, including brain, liver, cardiovascular, and pulmonary. This figure displays the relative percentages of intention-to-treat data of assessed risk of bias across human studies. The overall bias was determined using five categories: Selection of the reported result, measurement of the reported outcome, missing outcome data, deviations from intended interventions, and randomization process. Risk was determined as “low risk,” “some concerns,” or “high risk,” based on the Cochrane RoB 2.0 tool algorithm. The data for all human studies included 26 articles.

In assessing the risk of bias in human studies, we cannot ignore that the majority of human studies have been conducted in the heart and lungs, while limited data are available that address the role of IL-33 in the brain and liver. Nonetheless, in assessing risk of bias in all the human studies, 45% demonstrated some concerns while 75% demonstrated a high risk of bias (Figure 7). Fifty percent of studies demonstrated some concerns or a high risk of bias. Measurement of the intended outcome demonstrated the lowest risk of bias, while variability in levels of bias was most evident when assessing missing outcome data and deviations from intended interventions. Lastly, in humans, the designation of individual cell types as pro-inflammatory or regulatory has not been clearly outlined, as well as in murine models.

### 4.2. Conclusions/Limitations

IL-33 is differentially expressed in mice and humans. In addition, publicly available data sets may not agree in their assessment of IL-33 protein expression in human tissues. As an example, when we compared reported tissue protein expression of IL-33 in the GTEx, Human Protein Atlas, and ProteomicsDB databases (Table 6), there was no consensus regarding expression of IL-33 in the amygdala, basal ganglia, caudate, cerebellum, cerebral cortex, hippocampus, hypothalamus, and midbrain. There was also no consensus regarding IL-33 expression in liver, where no expression was reported in the Human Protein Atlas. Interestingly, there was consensus regarding expression of IL-33 in the heart and lung, which might suggest that these datasets may not have the most current data regarding known IL-33 expression in the brain and liver. It is clear that the discovery of the role of IL-33 in the specific context of various organs has opened an array of research opportunities to investigate the global or systemic effects of inflammation. It is also clear that there are several missing components in understanding IL-33 mechanisms. In assessing the risk of bias for each study, all studies displayed a probable or high risk of bias. A major source of bias across the studies was a lack of randomization. Furthermore, a potential risk of bias could also arise from the majority of studies focusing on mice, with the remainder focusing on humans or both mice and humans.

Among the organ systems, the brain displayed a potential risk of bias due to a greater number of studies focusing on the pro-inflammatory role of IL-33. The search strategy did not yield any articles focused exclusively on the effects of IL-33 in the liver in humans. All studies included for the liver were either mouse studies or review articles focused on both humans and mice. Within the heart, a majority of studies focused on the regulatory role of IL-33, which is a potential source of bias. Lastly, within the pulmonary system, there was a large discrepancy between articles that focused on mice and those that focused on humans, introducing another potential source of bias. Interestingly, no studies demonstrated a low risk of bias.

Several important gaps in the knowledge have been identified in our current knowledge and understanding of the role of IL-33 in the brain–liver connection. One potential gap in the knowledge is whether there is a significant effect of mouse background on the anxiety phenotype seen in IL-33 mice of a C57BL/6 background. It has already been previously shown that BALB/c mice display an increased level of anxiety-related behaviors as compared to C57BL/6 mice. In addition, BALB/c mice display a heightened stress response, with an elevated stress-induced plasma cortisol level following an acute stressor [16]. Previous studies have been done in mice with a C57BL/6 background; therefore, it is unknown what role IL-33 plays in affecting the brain–liver connection of BALB/c mice. Differences between BALB/c and C57BL/6 mice also include several factors of the immune response. BALB/c mice have displayed increased hepatic and plasma levels of cytokines and chemokines when compared to C57BL/6 mice [54]. In addition, there are differences in macrophage functions. Macrophages in C57BL/6 mice produce higher levels of TNF-alpha and IL-12 when compared to BALB/c mice [54]. At this point, it is unclear which mouse model is the most accurate reflection of the human central nervous system and liver. Further research needs to be done to determine which model, with all differences considered, is most generalizable to the human organ systems affected.

Within the central nervous system, previous studies have identified cytokine imbalances as having an involvement in anxiety-related behaviors and disorders. In particular, an increase in pro-inflammatory response and decrease in anti-inflammatory response have been associated with anxiety disorders, such as generalized anxiety disorder [55]. Therefore, the major gap is how the immune system and peripheral inflammation affect the brain. A future area of study involves identifying the liver as a surrogate marker. In this way, treating the brain will effectively treat problems in the periphery or vice versa. Along these lines, the majority of studies utilized mice with global IL-33 deficiency, while it is not clear whether organ-specific IL-33 deficiency will yield the same conclusions. Even so, it is not clear whether differential IL-33 expression in humans has a role in disease processes. Thus, many questions remain in terms of the role of the IL-33/ST2 axis in humans and in mice.

The identification of IL-33 as a prominent component of the immune response was an important discovery in better understanding systemic and organ-specific inflammation [56]. Although IL-33 has been well-characterized in cells within the central nervous and hepatic systems in murine models, the pro-inflammatory and regulatory mechanisms driving disease states in these organs are not well understood. More research is needed to assess the diagnostic or therapeutic potential of IL-33 in diseases affecting the central nervous system and the liver.

Supplementary materials consist of the Cochrane RoB 2.0 tool raw data utilized in this focused review (Excel file) as well as a list of references excluded from this review using criteria described in the Methods that may be useful to our readers (Word file).

## Figures and Tables

**Table 1:** Detailed keywords used in search strategy.

Population	Intervention	Comparison Intervention	Outcome Measures
C57BL/6 mice BALB/c mice Humans Murine model Central Nervous System Brain Liver Hepatic System Cardiovascular System Heart Cardiac Pulmonary System Lungs	IL-33 Interleukin-33 IL-1 Cytokine Family IL-10 IL-3 IL-5 Microglia Chemokines Cytokines Astrocytes Oligodendrocytes Neurons Sinusoidal endothelial cells Macrophages M2 Macrophages Hepatic stellate cells Human liver hepatocytes Cardiomyocytes Fibroblasts FoxP3+ Tregs Regulatory T Cells Endothelial cells Pulmonary endothelial cells ST2 receptor sST2 Th2 Cells ILC2s	C57BL/6 mice BALB/c mice Humans Murine model Central Nervous System Brain Liver Hepatic System Cardiovascular System Heart Cardiac	Pro-inflammatory Inflammatory Anti-inflammatory Regulatory Suppression

**Table 2:** IL-33 action in the brain.

Location	Action/Mechanism
Astrocytes	Constitutive IL-33 expression
Microglia	High expression of IL-33; pro-inflammatory response (TNF-α, IL-10, IL-1β, oxidative stress molecules)
Oligodendrocytes	Constitutive IL-33 expression
Neurons	Constitutive IL-33 expression

**Table 3:** IL-33 expression in various brain regions.

Brain Region(s)	IL-33 Expression
Corpus callosum & secondary motor cortex	Oligodendrocytes (Olig2+)
Medial prefrontal cortex	Oligodendrocytes and astrocytes (Olig2+ and S100β+)
Periventricular hypothalamic nucleus, basolateral amygdala, cortical amygdala	Astrocytes (S100β+)
Neurons & microglia	No expression

**Table 4:** IL-33 summary table (mice).

Location	Effect (Pro-inflammatory or Regulatory)
Central Nervous System	Pro-inflammatory roles: Microglia (via release of pro-inflammatory cytokines, chemokines, and oxidative stress molecules)
	Regulatory roles: Astrocytes, oligodendrocytes, neurons
Hepatic System	Pro-inflammatory roles: Sinusoidal endothelial cells (HSECs), macrophages, hepatic stellate cells (HSCs)
	Regulatory roles: FoxP3+ Tregs
Cardiovascular System	Pro-inflammatory roles: Endothelial cells
	Regulatory roles: Unknown
Pulmonary System	Pro-inflammatory roles: Pulmonary endothelial cells, macrophages, epithelial cells
	Regulatory roles: ST2 receptor, M2 macrophages

**Table 5:** IL-33 summary table (humans).

Location	Effect
Central Nervous System	Constitutive expression in glial cells (oligodendrocytes and astrocytes)
Hepatic System	Nuclear factor and inflammatory mediator; expressed in human liver hepatocytes (cytoplasm and nucleus)
Cardiovascular System	Constitutive expression in endothelial cells, cardiomyocytes, and fibroblasts
Pulmonary System	Pro-inflammatory role in lung epithelial cells

**Table 6:** Reported IL-33 protein expression in humans in the GTEx, Human Protein Atlas, and ProteomicsDB Databases.

Organ/Tissue	GTEx	Human Protein Atlas	ProteomicsDB
Adipose tissue	Y	Y	N
Adrenal gland	Y	N	N
Amygdala	Y	N	N
Appendix	N	Y	N
Basal ganglia	Y	N	N
Bone marrow	N	Y	N
Breast	Y	Y	N
Bronchus	N	Y	N
Caudate	N	Y	N
Cerebellum	Y	Y	N
Cerebral cortex	Y	Y	N
Cervix, uterine	Y	Y	Y
Colon	Y	Y	Y
Duodenum	N	N	N
Endometrium	Y	Y	N
Epididymis	N	N	N
Esophagus	Y	Y	Y
Fallopian tube	Y	N	N
Gallbladder	N	N	Y
Heart muscle	Y	Y	Y
Hippocampus	Y	Y	N
Hypothalamus	Y	N	N
Kidney	Y	N	N
Liver	Y	N	Y
Lung	Y	Y	Y
Lymph node	N	Y	Y
Midbrain	Y	N	N
Myometrium	N	N	Y
Nasopharynx	N	Y	N
Oral Mucosa	N	N	N
Ovary	Y	N	N
Pancreas	Y	Y	Y
Parathyroid gland	N	N	N
Pituitary gland	Y	N	N
Placenta	N	N	Y
Prostate	Y	Y	Y
Rectum	N	N	N
Salivary gland	Y	Y	N
Seminal vesicle	N	N	N
Skeletal muscle	Y	Y	N
Skin	Y	Y	Y
Small Intestine	Y	N	N
Smooth muscle	N	Y	N
Soft tissue	N	Y	N
Spinal cord	Y	N	N
Spleen	Y	Y	N
Stomach	Y	Y	N
Testis	Y	Y	Y
Thyroid gland	Y	N	N
Tonsil	N	Y	N
Urinary bladder	Y	Y	Y
Uterus	N	N	Y
Vagina	Y	Y	N

**Legend:** Y = Yes; N = No. The tissues highlighted in green belong to the brain and liver.

## Supplementary Materials

Supplementary materials can be found at https://www.mdpi.com/1422-0067/21/11/3933/s1; Cochrane RoB 2.0 tool raw data (Excel File); Supplementary references (Word file).
