# Supplementary material for: The Role of Pro-Inflammatory and Regulatory Signaling by IL-33 in the Brain and Liver: A Focused Systematic Review of Mouse and Human Data and Risk of Bias Assessment of the Literature
Source: Int J Mol Sci. 2020 May 30;21(11):3933. doi: 10.3390/ijms21113933 (PMC7312033; doi:10.3390/ijms21113933)
Supplement: Supplementary file 1 [file ijms-21-03933-s001.zip › ijms-805880 supplementary.docx]

**Supplementary References**

1. Altara, R.; Ghali, R.; Mallat, Z.; Cataliotti, A.; Booz, G.W.; Zouein, F.A. Conflicting vascular and metabolic impact of the IL-33/sST2 axis*. Cardiovasc. Res.* **2018**, *114*, 1578–1594.
2. Castanhinha, S.; Sherburn, R.; Walker, S.; Gupta, A.; Bossley, C.J.; Buckley, J.; Ullmann, N.; Grychtol, R.; Campbell, G.; Maglione, M.; et al. Pediatric severe asthma with fungal sensitization is mediated by steroid-resistant IL-33. *J. Allergy Clin. Immunol.* **2015**, *136*, 312–322, doi:10.1016/j.jaci.2015.01.016.
3. Chen, W.Y.; Tsai, T.H.; Yang, J.L.; Li, L.C. Therapeutic Strategies for Targeting IL-33/ST2 Signaling for the Treatment of Inflammatory Diseases. *Cell. Physiol. Biochem.* **2018**, *49*, 349–358.
4. de Grove, K.C.; Provoost, S.; Brusselle, G.; Joos, G.F.; Maes, T. Insights in particulate matter-induced allergic airway inflammation: Focus on the epithelium. *Clin. Exp. Allergy* **2018**, *48*, 773–786, doi:10.1111/cea.13178.
5. Gorska, K.; Nejman-Gryz, P.; Paplińska-Goryca, M.; Korczyński, P.; Prochorec-Sobieszek, M.; Krenke, R. Comparative Study of IL-33 and IL-6 Levels in Different Respiratory Samples in Mild-to-Moderate Asthma and Copd. *COPD J. Chronic Obstr. Pulm. Dis.* **2018**, *15*, 36–45, doi:10.1080/15412555.2017.1416074.
6. Hirahara, K.; Shinoda, K.; Morimoto, Y.; Kiuchi, M.; Aoki, A.; Kumagai, J.; Kokubo, K.; Nakayama, T. Immune Cell-Epithelial/Mesenchymal Interaction Contributing to Allergic Airway Inflammation Associated Pathology. *Front. Immunol.* **2019**, *10*, 570, doi:10.3389/fimmu.2019.00570.
7. Hong, J.; Kim, S.; Lin, P. Interleukin-33 and ST2 Signaling in Tumor Microenvironment. *J. Interf. Cytokine Res.* **2019**, *39*, 61–71, doi:10.1089/jir.2018.0044.
8. Hu, L.A.; Fu, Y.; Zhang, D.N.; Zhang, J. Serum IL-33 as a diagnostic and prognostic biomarker in non-small cell lung cancer. *Asian Pac. J. Cancer Prev.* **2013**, *14*, 2563–2566.
9. Johansson, K.; McSorley, H. Interleukin‐33 in the developing lung—Roles in asthma and infection. *Pediatr. Allergy Immunol.* **2019**, *30*, 503–510, doi:10.1111/pai.13040.
10. Lee, J.-U.; Chang, H.S.; Lee, H.J.; Jung, C.A.; Bae, D.J.; Song, H.J.; Park, J.S.; Uh, S.-T.; Kim, Y.H.; Seo, K.-H.; et al. Upregulation of interleukin-33 and thymic stromal lymphopoietin levels in the lungs of idiopathic pulmonary fibrosis. *BMC Pulm. Med.* **2017**, *17*, 39, doi:10.1186/s12890-017-0380-z.
11. Liu, T.; Kanaoka, Y.; Barrett, N.A.; Feng, C.; Garofalo, D.; Lai, J.; Buchheit, K.; Bhattacharya, N.; Laidlaw, T.M.; Katz, H.R.; et al. Aspirin-Exacerbated Respiratory Disease Involves a Cysteinyl Leukotriene-Driven IL-33-Mediated Mast Cell Activation Pathway. *J. Immunol.* **2015**, *195*, 3537–3545, doi:10.4049/jimmunol.1500905.
12. Majewski, S.; Tworek, D.; Szewczyk, K.; Kurmanowska, Z.; Antczak, A.; Gorski, P.; Piotrowski, W.J. Epithelial alarmin levels in exhaled breath condensate in patients with idiopathic pulmonary fibrosis: A pilot study. *Clin. Respir. J.* **2019**, *13*, 652–656, doi:10.1111/crj.13075.
13. Paplinska-Goryca, M.; Nejman-Gryz, P.; Górska, K.; Białek-Gosk, K.; Hermanowicz-Salamon, J.; Krenke, R. Expression of Inflammatory Mediators in Induced Sputum: Comparative Study in Asthma and COPD. *Plant Promot. Transc. Fac.* **2016**, *1040*, 101–112, doi:10.1007/5584_2016_165.
14. Roan, F.; Obata-Ninomiya, K.; Ziegler, S.F. Epithelial cell-derived cytokines: More than just signaling the alarm. *J. Clin. Investig.* **2019**, *129*, 1441–1451, doi:10.1172/JCI124606.
15. Rossios, C.; Pavlidis, S.; Hoda, U.; Kuo, C.; Wiegman, C.; Russell, K.; Sun, K.; Loza, M.J.; Baribaud, F.; Durham, A.L.; et al. Sputum transcriptomics reveal upregulation of IL-1 receptor family members in patients with severe asthma. *J. Allergy Clin. Immunol.* **2017**, *141*, 560–570.
16. Segiet, O.A.; Piecuch, A.; Mielańczyk, L.; Michalski, M.; Nowalany-Kozielska, E. Role of interleukins in heart failure with reduced ejection fraction. *Anatol. J. Cardiol.* **2019**, *22*, 287–299, doi:10.14744/anatoljcardiol.2019.32748.
17. Tseng, C.C.S.; Huibers, M.M.H.; van Kuik, J.; de Weger, R.A.; Vink, A.; de Jonge, N. The Interleukin-33/ST2 Pathway Is Expressed in the Failing Human Heart and Associated with Pro-fibrotic Remodeling of the Myocardium*. J. Cardiovasc. Trans. Res.* **2018**, *11*, 15–21.
18. Verheijden, K.; Willemsen, L.E.M.; Braber, S.; Leusink-Muis, T.; Delsing, D.J.M.; Garssen, J.; Kraneveld, A.D.; Folkerts, G. Dietary galacto-oligosaccharides prevent airway eosinophilia and hyperresponsiveness in a murine house dust mite-induced asthma model. *Respir. Res.* **2015**, *16*, 17, doi:10.1186/s12931-015-0171-0.
